# Sublethal cadmium exposure disrupts cardiac function and tissue structure in zebrafish larvae

**DOI:** 10.3389/fphys.2026.1865285

**Published:** 2026-09-21

**Authors:** Alexander Machikhin, Anastasia Guryleva, Viacheslav Krylov, Valeriya Bukova, Artem Slavin, Leonid Shuman, Alexander Selyukov, Alexander Burlakov, Anirban Chakraborty

**Affiliations:** 1 Acousto-optic Spectroscopy Lab, Scientific and Technological Center of Unique Instrumentation, Russian Academy of Sciences, Moscow, Russia; 2 Population Biology and Genetics Lab, Papanin Institute for Biology of Inland Waters, Russian Academy of Sciences, Borok, Russia; 3 Department of Zoology and Evolutionary Ecology of Animals, University of Tyumen, Tyumen, Russia; 4 Department of Ichthyology, Faculty of Biology, Lomonosov Moscow State University, Moscow, Russia; 5 Nitte (Deemed to be University), Nitte University Centre for Science Education & Research, Department of Molecular Genetics & Cancer, Mangalore, India; 6 Nitte (Deemed to be University), KS Hegde Medical Academy, Department of Medical Oncology, Mangalore, India

**Keywords:** cadmium, cardiac activity, developmental abnormalities, histopathology, photoplethysmography, toxicity, zebrafish

## Abstract

Cadmium remains a primary toxicological concern because it does not degrade and easily enters the food chain, affecting diverse ecosystems. This study aimed to evaluate the effects of sublethal cadmium exposure on cardiac function and on brain and heart tissues in zebrafish (*Danio rerio*) larvae, utilizing photoplethysmography as a rapid, non-invasive approach. Larvae (1 day post-hatching) were exposed for five days to cadmium sulfate at concentrations of 0.005, 0.025, and 0.05 mg/L under controlled laboratory conditions. Cardiac activity was assessed twice a day using a non-invasive photoplethysmography-based technique. Histological analyses of brain and heart tissues were conducted using standard fixation, sectioning, and staining protocols. At the highest cadmium concentration, histological examination revealed neuronal degeneration in brain tissue and thinning of the cardiac wall. Cadmium exposure led to heart rate suppression, with the most pronounced effect observed at 0.05 mg/L. Lower concentrations exhibited attenuated but still significant effects compared to controls. These findings indicate that cadmium exerts both functional and structural toxicity in zebrafish larvae, affecting cardiovascular development and neural integrity. The results support the use of heart rate, measured via photoplethysmography, as a sensitive and non-invasive endpoint for detecting sublethal cardiotoxic effects.

## 1 Introduction

Environmental contamination by heavy metals remains a pressing global concern. Cadmium is recognized as one of the most toxic and persistent pollutants among them. Naturally, cadmium enters the environment through geogenic processes such as the oxidation of mineralized rocks, river sediments, volcanic materials, and soils (Kubier et al., 2019). However, anthropogenic factors have led to a significant escalation of its concentration within the environment (McLaughlin et al., 2021; Sattar et al., 2025). Some of the common anthropogenic factors include industrial and municipal waste, hospital effluents, fertilizers, construction debris, paints and pigments, batteries, steel manufacturing, and cigarette smoke (Kubier et al., 2019; Zhang and Reynolds, 2019).

The accumulation of cadmium in ecosystems impacts nearly all life forms negatively (Gao et al., 2013; Losada Ros et al., 2020; Mo et al., 2022). Moreover, cadmium exhibits strong tendencies toward bioaccumulation and biomagnification, with its concentration increasing progressively along trophic levels (Lee et al., 2024). Consequently, top-level consumers, including humans, face a significant health threat from cadmium contamination (Kalahasthi et al., 2025; Zhang et al., 2025).

Standardized bioassays using model organisms are employed to assess the ecological impact of cadmium. One such prominent organism is the zebrafish (*Danio rerio*), which has become an indispensable vertebrate model for toxicological research. Numerous studies have documented the harmful effects of cadmium exposure in zebrafish (Okutsu et al., 2025). In recent years, non-invasive assessment of heart rate (HR) in transparent zebrafish embryos and larvae has gained traction as a rapid and sensitive approach for detecting toxicant-induced stress (Meng et al., 2020; Krylov et al., 2025; Liu et al., 2026), including cadmium exposure (Wold et al., 2017; Lin et al., 2023; Heuer et al., 2024). This method is particularly valuable because it provides a rapid biological response to a toxicant exposure.

In addition to cardiotoxicity, cadmium is widely recognized as a potent neurotoxicant capable of disrupting normal brain development and inducing structural alterations in neural tissues. Previous studies have reported that cadmium exposure in zebrafish can lead to oxidative stress, neuronal degeneration, impaired neurogenesis, and abnormalities in central nervous system development (Monaco et al., 2017). Because neural regulation plays a crucial role in cardiovascular function, cadmium-induced changes in heart rate may partially reflect disruptions in nervous system development rather than isolated cardiac effects alone. Therefore, evaluating histopathological changes in brain tissue along with cardiac endpoints may provide a more comprehensive understanding of cadmium toxicity.

Nevertheless, reports on cadmium’s impact on zebrafish cardiac activity remain inconsistent; while some studies described a decrease in HR (Heuer et al., 2024), others observed an increase (Wold et al., 2017). These discrepancies underscore the need for further investigation of cadmium-induced cardiotoxicity and its relationship with damage to other vulnerable organs, particularly the developing nervous system. Therefore, this study aimed to evaluate the effects of cadmium exposure on cardiac function as well as morphological alterations in brain and heart tissue of zebrafish larvae. This study utilizes photoplethysmography as a rapid, non-invasive approach for detecting sublethal cardiotoxic effects during early zebrafish development, complemented by histological validation.

## 2 Materials and methods

### 2.1 Animals

Wild-type zebrafish (*D. rerio*, AB strain) were obtained from a commercial distributor. Husbandry and experimental procedures followed established ZFIN guidelines (Westerfield, 2007). We kept zebrafish at 27 ˚C under a 16 h light/8 h dark cycle. Water parameters were maintained as follows: dissolved oxygen ~7.5 mg/L, conductivity ~388 mS/cm, hardness ~10.95 dGH, and pH ~7.3.

All experimental procedures complied with relevant guidelines and were approved by the Bioethics Committee of the Scientific and Technological Centre of Unique Instrumentation of the Russian Academy of Sciences (protocol No. 3/24, dated 21 August 2024).

### 2.2 Experimental protocol

A total of 48 zebrafish larvae obtained from a single spawning were used in each experimental replication. At ~72 hours post-fertilization (1 day post-hatching), larvae were randomly assigned to four experimental groups (8 individuals per group within each replicate). Larvae were placed individually into 24-well plates containing 2.5 mL of either control medium or cadmium sulfate solutions at concentrations of 0.005, 0.025, or 0.05 mg/L. The control medium consisted of cultivation water, which was also used for preparing all cadmium solutions.

Larvae remained exposed to cadmium for 5 days. The control medium and cadmium solutions were completely renewed daily. Heart rate was measured once on Day 0 (after transfer into the exposure wells) and twice daily on Days 1–4 (in the morning and evening). Morning sessions were conducted between 09:00 and 12:00 and evening sessions between 16:00 and 19:00. Each treatment was performed in three independent replicates, with a planned sample size of 24 larvae per group (3 replicates × 8 larvae). Only recordings with an analyzable photoplethysmographic signal were included in **Supplementary Table 1**; therefore, n varied among days, groups, and sessions.

Developmental stage was additionally assessed visually from bright-field images to evaluate the potential effects of cadmium exposure on the rate of early development. Representative images of larvae from different groups on the first and final days of the experiment are shown in **Supplementary Figure 6**. Comparison of the principal morphological markers of developmental progression in zebrafish, including body length, the degree of swim bladder inflation, pericardial area, and yolk sac resorption, indicated that the rate of early development was comparable between the control and experimental groups. After the final HR measurement on day 4, four larvae from each group within each replicate were randomly selected and fixed for histological analysis (n = 12 per group across all replicates).

### 2.3 Photoplethysmography

Cardiac activity was assessed using a non-invasive photoplethysmography-based technique for monitoring temporal changes in tissue blood flow. This method has previously been validated for HR measurements in zebrafish embryos and larvae (Machikhin et al., 2020; Krylov et al., 2025).

Image sequences of cardiac activity were acquired using an Altami Bio6 microscope equipped with a 4× PLAN APO lens and a monochrome camera (The Imaging Source DMK 33UX252; maximum resolution 2048 × 1535 pixels; pixel size 3.45 µm; up to 120 Hz). Each larva was transferred with a plastic pipette into a 35 mm Petri dish containing anesthetic solution, immobilized for approximately 1 min, and positioned in lateral recumbency on the microscope stage to visualize the cardiac region through the transparent body wall; gentle repositioning with a pipette tip was used when needed. Groups were processed in a rotating order across sessions to avoid systematic time-of-day bias among treatments.

Image sequences (12-bit, 1000 × 1000 pixels) were recorded for 20 s at 50 Hz. The recordings were processed using previously established algorithms (Machikhin et al., 2022; Krylov et al., 2024; Sergeeva et al., 2025) to extract photoplethysmographic signals that reflect pixel-intensity fluctuations associated with cardiac activity. HR was derived from these photoplethysmograms. The processing pipeline included image contrast enhancement, frame-by-frame image alignment for motion correction, calculation of signal standard deviation, identification of the cardiac region based on maximal signal variance, averaging of pixel intensities within the identified region for each frame, and band-pass filtering of the resulting signal within the 0.5–7 Hz range using Fourier transform.

After the 20 s recording, each larva was returned to clean exposure solution matching its assigned group (control medium or the corresponding cadmium concentration) without anesthetic. Using a Pasteur pipette with the tip trimmed to approximately 5 mm in diameter, the larva was rinsed in clean solution, held for 5 min, and returned to its original well. The same individuals were measured over time during exposure, except when a recording was excluded because the larva left the field of view or the signal could not be analyzed after motion correction and filtering. Imaging was performed in a room held at the same temperature as the plates. The temperature in the Petri dish was monitored with an immersed thermocouple-based sensor. It remained within 0.5 °C of the plate’s temperature. Before the experiments, preliminary measurements of oxygen levels in the Petri dish water were taken before and after the fish was held in the dish for one minute. No significant changes were recorded, as the dish offers a relatively large surface area for gas exchange.

### 2.4 Chemicals

Cadmium sulfate solutions (0.005, 0.025, and 0.05 mg/L) were prepared by diluting a 98% purity stock (Sigma-Aldrich, USA) in cultivation water (pH 7.3 ± 0.1). These concentrations were selected because prior studies indicated they could influence cardiac function without causing acute toxicity (Aldavood et al., 2020). The stock solution concentration was 0.5 mg/L. Working solutions were freshly prepared daily by dilution with cultivation water.

Direct analytical quantification of cadmium concentrations in the exposure media was not available in the present study. Nevertheless, the use of high-purity cadmium sulfate, precise gravimetric preparation procedures and daily renewal of exposure solutions suggest that nominal concentrations approximated actual concentrations throughout the experiment. However, the actual dissolved cadmium concentration may deviate from nominal values due to potential adsorption by container surfaces, complexation with constituents of the exposure medium, and uptake by developing organisms over the course of the exposure.

Buffered tricaine methanesulfonate (MS-222) was used at a concentration of 0.168 mg/L in accordance with ZFIN recommendations for anesthesia before cardiac measurements. Standardization of MS-222 application, including anesthetic concentration and exposure duration, minimized potential bias from anesthesia and ensured comparability of HR measurements across experimental groups.

### 2.5 Histology

For histological analysis, 12 larvae per group were fixed in Bouin’s solution (75% picric acid, 10% formalin, 5% acetic acid; pH 1.0). After fixation, samples were thoroughly washed in 70% ethanol to remove excess picric acid, minimizing additional hardening. Samples were dehydrated through a graded ethanol series, embedded in paraffin (EC-350, Thermo Scientific Microm), sectioned using an HM 355S microtome (Thermo Scientific Microm) at a thickness of 5 µm, and stained with Heidenhain’s AZAN trichrome.

Larval sections were analyzed using an AxioImager A1 microscope. Quantitative measurements were performed using AxioVision software (Carl Zeiss, AxioVision Rel. 4.8). The assessment of zebrafish larval heart wall thickness on paraffin sections was conducted using a well-established method (Hu et al., 2000; Qian et al., 2025). The thickness of atrial and ventricular walls was measured in each larva at three different locations on each of three sagittal sections passing through the central region of the atrium and ventricle. Sections passing through the lateral regions of the heart were excluded from analysis (Hu et al., 2000).

Microscopic analysis additionally revealed areas of degenerative neural tissue in larval brains. It was assessed on three histological sections of each zebrafish larval brain tissue based on a combination of morphological criteria, including the presence of pyknotic nuclei, cellular shrinkage, increased cytoplasmic eosinophilia, and disruption of normal tissue architecture compared to control specimens (Wu et al., 2025). Degenerating neurons were identified within defined brain regions and quantified as the proportion of affected cells relative to the total number of morphologically intact neurons in the same field of view.

To minimize observer bias, all histological evaluations were performed in a blinded manner. The researcher responsible for image analysis was unaware of experimental treatment for each specimen during data acquisition and quantification. Group identities were revealed only after completion of all measurements.

### 2.6 Statistical analysis

Differences in HR among groups were evaluated separately for each measurement session using one-way ANOVA followed by Tukey’s *post hoc* test (p < 0.05). Normality and homogeneity of variances were verified before analysis. Descriptive statistics (n, mean ± SD), ANOVA results, and pairwise *post hoc* contrasts are given in **Supplementary Table 5**. Spearman rank correlation between nominal cadmium concentration and individual HR values was calculated for each session; coefficients and p-values are given in **Supplementary Table 6**.

Differences in the size of regions of neuronal degeneration in the brain were assessed using the Mann–Whitney U test because the data were not normally distributed. Bonferroni correction was applied to adjust for multiple comparisons, with the significance threshold set at p < 0.017.

## 3 Results

The applied cadmium exposures did not significantly affect the survival of zebrafish larvae. No mortalities were observed during five experimental days.

Histological analysis of neural tissue revealed that larvae exposed to 0.05 mg/L cadmium exhibited localized regions of neuronal degeneration occupying 5.33 ± 3.66% of the tissue section area, significantly higher than in controls (0.05 ± 0.13%; p < 0.001; **Figures 1A, B, F**; **Supplementary Table 4**). At 0.025 mg/L, the mean proportion was 0.21 ± 0.26% and did not differ from the control group (p = 0.40; **Supplementary Table 4**).

**Figure 1:**
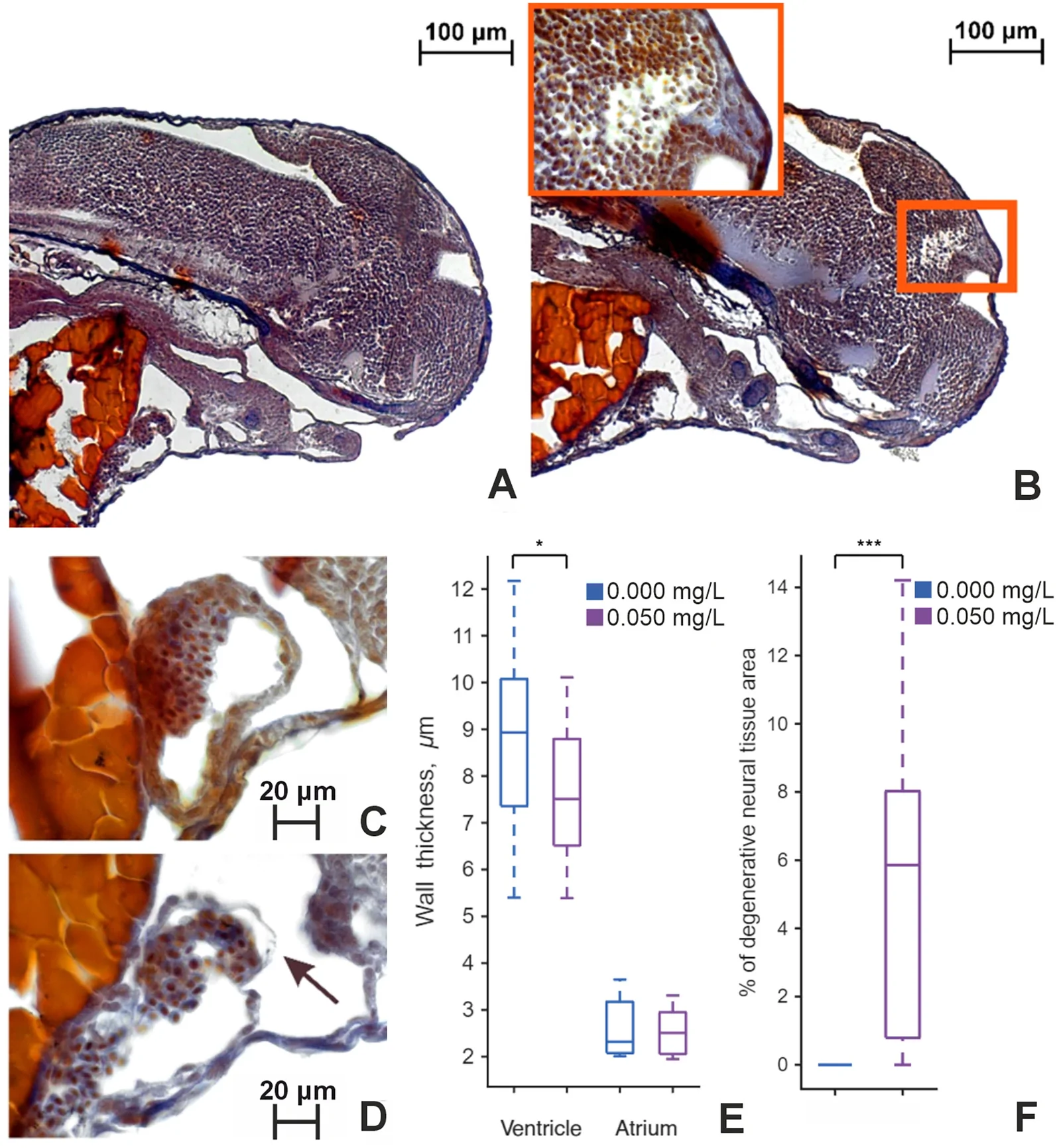
Histological alterations in zebrafish tissues: **(A)** brain, control group; **(B)** brain, exposed to 0.05 mg/L cadmium; **(C)** cardiac region, control group; **(D)** cardiac region, exposed to 0.05 mg/L cadmium; the arrow indicates thinning of the cardiac wall; **(E)** ventricular and atrial wall thickness in control and 0.05 mg/L larvae; **(F)** proportion of neuronal degeneration in control and 0.05 mg/L groups. In boxplots, the central line indicates the median; the lower and upper boundaries of the box represent the 25th and 75th percentiles, respectively; whiskers extend to the most extreme data points; and asterisks denote significant differences relative to the control group (*p < 0.05; ***p < 0.001).

Examination of cardiac tissue showed thinning of the ventricular myocardial wall in larvae exposed to 0.05 mg/L cadmium compared with controls (**Figures 1C–E**; **Supplementary Table 3**): mean ventricular wall thickness was 8.75 µm in control larvae and 7.44 µm at 0.05 mg/L (p < 0.05).

Neuronal degeneration morphometry was not performed for the 0.005 mg/L group, and cardiac wall thickness was not quantified morphometrically for the 0.005 and 0.025 mg/L groups because the available histological sections did not contain the anatomical regions required for these measurements. This resulted from technical inaccuracies during sample preparation. We therefore had to choose between excluding these histological data entirely or retaining the measurements obtained from the available sections. During an earlier stage of the manuscript review process, we concluded that these partial data were nevertheless informative and therefore retained them in the manuscript.

Heart rate changes during exposure are shown in **Figure 2** and **Supplementary Table 2**. In the control group, mean HR increased progressively over the first four days of development from 159 ± 13 bpm on Day 0 to 226 ± 36 bpm on Day 4; all consecutive-day comparisons were significant (Days 0-1, 1-2, 2-3, and 3-4: p = 0.006, 0.022, 0.030, and <0.0001, respectively). At 0.005 mg/L, HR changed from 165 ± 8 to 183 ± 28 bpm; Days 0-1, 1-2, and 2–3 were not significant (p = 0.84, 0.53, and 0.77), whereas Day 3–4 was significant (p = 0.0006). At 0.025 mg/L, HR increased from 139 ± 19 to 160 ± 26 bpm; Day 0–1 was not significant (p = 0.41), whereas Days 1-2, 2-3, and 3–4 were significant (p < 0.0001, 0.010, and 0.034, respectively). At 0.05 mg/L, HR changed from 111 ± 11 to 122 ± 15 bpm; only Day 0–1 was significant (p = 0.011), whereas later intervals were not (Days 1-2, 2-3, and 3-4: p = 0.52, 0.19, and 0.90, respectively). One-way ANOVA showed significant differences among groups in every session (all p < 0.001; **Supplementary Table 5**), with the largest suppression at 0.05 mg/L (**Figure 2**; **Supplementary Table 5**). Spearman correlations between cadmium concentration and HR were negative in all sessions (ρ = -0.67 to -0.88, all p < 0.001; **Supplementary Table 6**). Cadmium exposure therefore suppressed the normal developmental increase in HR.

**Figure 2:**
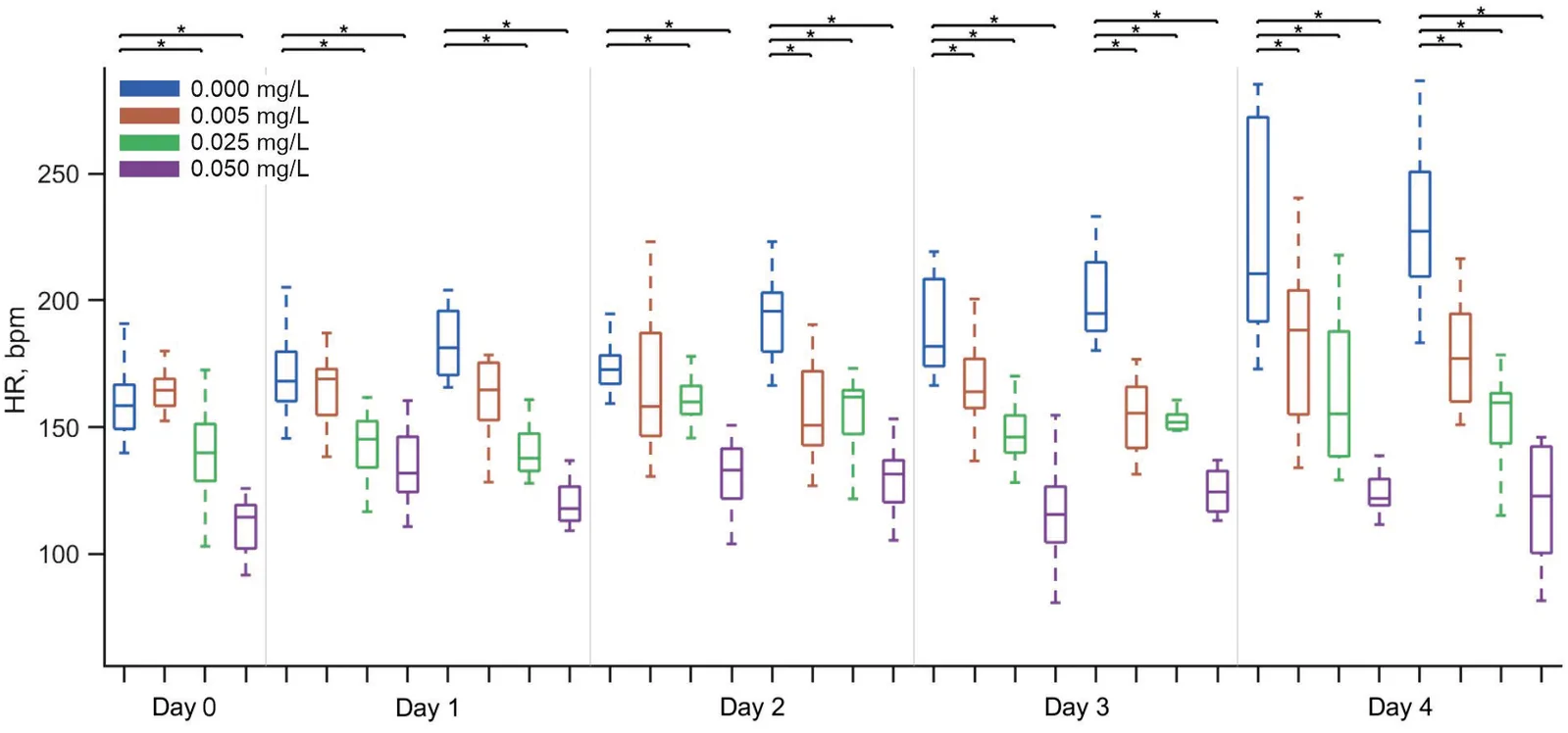
Boxplots showing daily heart rate distributions across experimental groups. The central line indicates the median; the lower and upper boundaries of the box represent the 25th and 75th percentiles, respectively; whiskers extend to the most extreme data points; and asterisks denote significant differences relative to the control group (*p < 0.05).

## 4 Discussion

It is essential to emphasize that embryonic and larvae stages are the most susceptible and vulnerable to toxicant exposure, including cadmium (Modarresi Chahardehi et al., 2020; Manna and Firdous, 2025). This heightened sensitivity is associated with the incomplete development of detoxification systems and the high permeability of tissues (Wiegand et al., 2000). Disruptions arising at early stages of life may be irreversible and can lead to severe consequences for subsequent organismal development (Hawkey et al., 2023).

Our findings are consistent with previously published studies that report significant reductions in HR following exposure to sublethal cadmium concentrations. For example, Mitovic et al. (2021) demonstrated that zebrafish embryos exposed to sublethal Cd²_+_ concentrations exhibited a 13.1% reduction in HR (113 ± 9 bpm) compared with controls (130 ± 7 bpm). Similarly, Sugiyono et al. (2026) reported that exposure to 1 or 10 μM CdCl_2_ significantly decreased HR at 72 h post-fertilization. Heuer et al. (2024) also observed approximately 11.6% lower HR in zebrafish larvae exposed to 5 μg/L cadmium relative to controls. In the present study, significant reductions in HR ranged from 19.1% to 46.1%, depending on cadmium concentration and exposure duration. These observations indicate that suppression of cardiac activity is a reproducible response of developing zebrafish to cadmium exposure.

Changes in HR in zebrafish embryos and larvae exposed to cadmium are likely associated with a complex set of toxic effects exerted by this heavy metal. Although mechanistic pathways were not directly investigated in the present study, previous evidence suggests several plausible explanations. Cadmium has been shown to interfere with calcium channels in cardiomyocytes, disrupting calcium channels essential for excitation–contraction coupling and myocardial function (Mitovic et al., 2021). Cadmium triggers the generation of reactive oxygen species, which results in oxidative stress within cardiomyocytes, damages the mitochondrial membrane, impairs oxidative phosphorylation, and reduces the ATP production necessary for proper cardiac function (Đukić-Ćosić et al., 2020). Previous studies have also reported significant increase in oxidative stress markers in cardiac tissue following cadmium exposure, including increased lipid peroxidation and decreased activity of antioxidant enzymes (Soares et al., 2008).

Our findings also indicate that cadmium-induced alterations in HR are associated with structural and developmental abnormalities of the heart. These findings are consistent with those of (Mitovic et al., 2021), who showed that functional changes in the cardiovascular system due to cadmium exposure form part of a broader spectrum of developmental disturbances, including enlargement of the pericardial sac. The myocardial thinning observed in the present study may potentially reflect oxidative stress-induced cellular damage or apoptosis in cardiomyocytes (Chen et al., 2015; Đukić-Ćosić et al., 2020), although no markers of apoptosis were evaluated. Moreover, the effects of cadmium are systemic. An increase in apoptotic events has been reported in the brains of zebrafish following exposure to cadmium chloride (Monaco et al., 2017). This finding is broadly consistent with the degenerative neural alterations observed in our histological analysis. While our data do not suggest a direct relationship between neurodegeneration and heart rate, it can be hypothesized that these cerebral changes may exacerbate the cardiotoxicity of cadmium (Mann et al., 2010; Stoyek et al., 2016; Valenza et al., 2025).

The concentration-dependent effects of cadmium on heart rate in zebrafish observed in this study are also consistent with established toxicological patterns. Hallare et al. (2005) demonstrated that higher cadmium concentrations cause a gradual decline in cardiac function. Reported LC_50_ values for cadmium sulfate in zebrafish embryos, expressed in terms of cadmium ions, are on the order of 20 mg/L (Ali et al., 2011; Fu et al., 2025). Notably, HR responses observed in the present study occurred at concentrations several orders of magnitude below LC_50_. The cadmium concentrations used in this study are environmentally relevant. Previous studies reported cadmium concentrations approaching or exceeding 0.005 mg/L in surface waters from certain contaminated locations (Bandara et al., 2011; Mu et al., 2014). Therefore, non-invasive HR assessment using photoplethysmography may represent a sensitive endpoint for environmental monitoring and heavy metal contamination risk assessment, suggesting that cardiac activity may respond to environmentally relevant exposure levels before overt lethality develops. Nevertheless, broader application of this biomarker requires validation across multiple toxicants and stressors to determine its specificity and robustness.

Overall, the results expand current understanding of cadmium cardiotoxicity and highlight the need for further studies to elucidate its underlying mechanisms, as well as to develop effective approaches for monitoring and mitigating its impact on aquatic ecosystems. The optical transparency of zebrafish embryos and larvae, along with advanced imaging techniques, makes this model particularly well-suited for cardiotoxicity studies. Future work should focus on validating HR reaction across diverse toxicants, integrating molecular and histological endpoints, developing automated analytical pipelines incorporating artificial intelligence approaches for high-throughput toxicological screening.

## Data Availability

The raw data supporting the conclusions of this article will be made available by the authors, without undue reservation.

## Supplementary material

Supplementary Figure 1 Temporal dynamics of average daily heart rate values in cadmium-exposed and control groups. Data are presented as mean ± standard error of the mean (SEM).

Supplementary Figure 2 Original image from **Figure 2A**.

Supplementary Figure 3 Original image from **Figure 2B**.

Supplementary Figure 4 Original image from **Figure 2C**.

Supplementary Figure 5 Original image from **Figure 2D**.

Supplementary Figure 6 Larvae of different experimental groups on Day 0 and Day 4
