# Supplementary material for: Sublethal cadmium exposure disrupts cardiac function and tissue structure in zebrafish larvae
Source: Front Physiol. 2026 Sep 21;17:1865285. doi: 10.3389/fphys.2026.1865285 (PMC13634864; doi:10.3389/fphys.2026.1865285)
Supplement: Supplementary file 1 [file Table1.pdf]

Table S1

Day	Concentration	Repetition	HeartRate, bpm
0	0	1	153.8
0	0	1	157.2
0	0	1	160.1
0	0	1	190.8
0	0	1	162.3
0	0	1	159.8
0	0	1	173.0
0	0	1	139.8
0	0	1	149.3
0	0	1	166.7
0	0	1	171.6
0	0	1	142.2
0	0	1	149.1
0	0	1	153.2
0	0.005	1	152.4
0	0.005	1	166.6
0	0.005	1	164.3
0	0.005	1	159.0
0	0.005	1	158.2
0	0.005	1	169.5
0	0.005	1	164.6
0	0.005	1	176.9
0	0.005	1	180.0
0	0.005	1	158.2
0	0.005	1	167.7
0	0.025	1	128.5
0	0.025	1	128.8
0	0.025	1	132.3
0	0.025	1	149.6
0	0.025	1	156.1
0	0.025	1	131.4
0	0.025	1	140.3
0	0.025	1	102.9
0	0.025	1	116.3
0	0.025	1	108.6
0	0.025	1	164.1
0	0.025	1	172.5
0	0.025	1	139.8
0	0.025	1	161.2
0	0.025	1	138.1
0	0.025	1	148.1
0	0.025	1	148.4
0	0.05	1	104.0
0	0.05	1	96.4
0	0.05	1	118.9
0	0.05	1	91.6
0	0.05	1	114.5
0	0.05	1	115.6

0	0.05	1	120.4
0	0.05	1	108.5
0	0.05	1	125.8
1	0	1	178.2
1	0	1	181.8
1	0	1	191.5
1	0	1	159.8
1	0	1	183.8
1	0	1	176.7
1	0	1	181.3
1	0	1	205.2
1	0	1	169.2
1	0	1	162.1
1	0	1	145.5
1	0	1	164.5
1	0	1	167.0
1	0	1	178.2
1	0	1	177.5
1	0	1	156.1
1	0	1	164.3
1	0	1	157.8
1	0	1	160.7
1	0	1	147.5
1	0.005	1	173.6
1	0.005	1	138.3
1	0.005	1	165.2
1	0.005	1	182.8
1	0.005	1	181.8
1	0.005	1	171.6
1	0.005	1	180.8
1	0.005	1	186.5
1	0.005	1	187.1
1	0.005	1	146.6
1	0.005	1	169.5
1	0.005	1	169.5
1	0.005	1	149.5
1	0.005	1	146.0
1	0.005	1	154.7
1	0.005	1	172.9
1	0.005	1	149.7
1	0.005	1	156.6
1	0.005	1	155.1
1	0.005	1	166.8
1	0.005	1	153.9
1	0.005	1	161.8
1	0.005	1	169.9
1	0.005	1	168.5
1	0.025	1	151.1
1	0.025	1	138.7
1	0.025	1	143.9

1	0.025	1	133.4
1	0.025	1	134.3
1	0.025	1	151.9
1	0.025	1	135.2
1	0.025	1	134.3
1	0.025	1	116.6
1	0.025	1	130.7
1	0.025	1	136.1
1	0.025	1	132.9
1	0.025	1	160.4
1	0.025	1	160.1
1	0.025	1	151.6
1	0.025	1	148.7
1	0.025	1	132.5
1	0.025	1	133.0
1	0.025	1	150.1
1	0.025	1	147.0
1	0.025	1	161.7
1	0.025	1	156.0
1	0.025	1	153.7
1	0.025	1	157.6
1	0.05	1	150.7
1	0.05	1	125.0
1	0.05	1	131.8
1	0.05	1	110.8
1	0.05	1	124.2
1	0.05	1	160.5
1	0.05	1	132.7
1	0	2	165.7
1	0	2	170.6
1	0	2	195.8
1	0	2	204.1
1	0	2	181.3
1	0	2	181.3
1	0.005	2	159.3
1	0.005	2	178.4
1	0.005	2	172.4
1	0.005	2	151.0
1	0.005	2	146.9
1	0.005	2	154.6
1	0.005	2	164.5
1	0.005	2	164.8
1	0.005	2	174.5
1	0.005	2	128.3
1	0.005	2	176.3
1	0.005	2	268.5
1	0.025	2	160.8
1	0.025	2	134.4
1	0.025	2	132.7
1	0.025	2	127.9

1	0.025	2	151.3
1	0.025	2	127.8
1	0.025	2	135.5
1	0.025	2	139.9
1	0.025	2	147.4
1	0.025	2	146.5
1	0.05	2	113.2
1	0.05	2	111.3
1	0.05	2	113.0
1	0.05	2	116.1
1	0.05	2	119.6
1	0.05	2	125.4
1	0.05	2	136.8
1	0.05	2	127.5
1	0.05	2	130.7
1	0.05	2	123.5
1	0.05	2	114.0
1	0.05	2	109.1
2	0	1	165.1
2	0	1	167.1
2	0	1	177.6
2	0	1	166.5
2	0	1	159.3
2	0	1	171.5
2	0	1	176.0
2	0	1	173.9
2	0	1	171.5
2	0	1	168.7
2	0	1	186.0
2	0	1	194.7
2	0	1	186.7
2	0	1	178.3
2	0.005	1	132.4
2	0.005	1	134.1
2	0.005	1	130.5
2	0.005	1	185.1
2	0.005	1	193.2
2	0.005	1	150.8
2	0.005	1	156.7
2	0.005	1	150.7
2	0.005	1	158.1
2	0.005	1	209.6
2	0.005	1	223.1
2	0.005	1	175.7
2	0.005	1	181.9
2	0.025	1	164.0
2	0.025	1	172.5
2	0.025	1	130.0
2	0.025	1	147.1
2	0.025	1	156.3

2	0.025	1	158.4
2	0.025	1	157.2
2	0.025	1	165.5
2	0.025	1	165.8
2	0.025	1	160.0
2	0.025	1	159.9
2	0.025	1	167.4
2	0.025	1	183.1
2	0.025	1	158.8
2	0.025	1	151.7
2	0.025	1	145.7
2	0.025	1	177.9
2	0.05	1	128.3
2	0.05	1	113.5
2	0.05	1	121.8
2	0.05	1	124.8
2	0.05	1	126.0
2	0.05	1	137.9
2	0.05	1	143.2
2	0.05	1	140.9
2	0.05	1	137.8
2	0.05	1	141.5
2	0.05	1	103.9
2	0.05	1	142.3
2	0.05	1	66.5
2	0.05	1	150.7
2	0	2	179.8
2	0	2	182.7
2	0	2	169.6
2	0	2	166.4
2	0	2	203.0
2	0	2	176.5
2	0	2	247.9
2	0	2	191.1
2	0	2	200.4
2	0	2	201.8
2	0	2	180.3
2	0	2	201.6
2	0	2	223.2
2	0	2	209.1
2	0.005	2	144.1
2	0.005	2	147.6
2	0.005	2	147.9
2	0.005	2	153.5
2	0.005	2	139.8
2	0.005	2	141.6
2	0.005	2	126.8
2	0.005	2	174.0
2	0.005	2	160.5
2	0.005	2	170.1

2	0.005	2	190.0
2	0.005	2	190.4
2	0.025	2	160.2
2	0.025	2	164.6
2	0.025	2	161.7
2	0.025	2	162.0
2	0.025	2	121.7
2	0.025	2	133.9
2	0.025	2	118.1
2	0.025	2	147.3
2	0.025	2	163.6
2	0.025	2	164.7
2	0.025	2	168.4
2	0.025	2	173.2
2	0.025	2	163.6
2	0.025	2	158.8
2	0.05	2	131.2
2	0.05	2	105.3
2	0.05	2	115.0
2	0.05	2	133.6
2	0.05	2	122.7
2	0.05	2	153.2
2	0.05	2	118.2
2	0.05	2	121.1
2	0.05	2	140.9
2	0.05	2	142.0
2	0.05	2	131.5
2	0.05	2	131.8
2	0.05	2	135.6
3	0	1	173.0
3	0	1	177.2
3	0	1	166.4
3	0	1	178.4
3	0	1	181.9
3	0	1	186.6
3	0	1	172.9
3	0	1	169.9
3	0	1	179.3
3	0	1	219.2
3	0	1	196.9
3	0	1	218.9
3	0	1	199.4
3	0	1	211.4
3	0	1	215.8
3	0.005	1	197.2
3	0.005	1	200.5
3	0.005	1	171.5
3	0.005	1	182.7
3	0.005	1	167.2
3	0.005	1	154.1

3	0.005	1	157.9
3	0.005	1	156.5
3	0.005	1	136.6
3	0.005	1	160.6
3	0.005	1	163.7
3	0.005	1	163.9
3	0.005	1	174.9
3	0.025	1	100.1
3	0.025	1	105.0
3	0.025	1	144.6
3	0.025	1	146.1
3	0.025	1	155.9
3	0.025	1	143.9
3	0.025	1	170.1
3	0.025	1	161.2
3	0.025	1	154.2
3	0.025	1	146.6
3	0.025	1	143.9
3	0.025	1	148.2
3	0.025	1	128.1
3	0.05	1	125.6
3	0.05	1	149.3
3	0.05	1	152.1
3	0.05	1	154.7
3	0.05	1	88.6
3	0.05	1	99.4
3	0.05	1	89.9
3	0.05	1	80.7
3	0.05	1	109.6
3	0.05	1	115.1
3	0.05	1	117.9
3	0.05	1	116.0
3	0.05	1	110.8
3	0.05	1	118.4
3	0.05	1	127.5
3	0.05	1	112.4
3	0	2	216.3
3	0	2	204.3
3	0	2	218.9
3	0	2	233.1
3	0	2	226.5
3	0	2	214.6
3	0	2	213.9
3	0	2	182.4
3	0	2	194.8
3	0	2	192.7
3	0	2	180.2
3	0	2	202.7
3	0	2	186.5
3	0	2	190.8

3	0	2	182.6
3	0	2	188.5
3	0	2	189.7
3	0.005	2	141.6
3	0.005	2	137.9
3	0.005	2	140.2
3	0.005	2	142.4
3	0.005	2	145.6
3	0.005	2	151.7
3	0.005	2	156.9
3	0.005	2	176.7
3	0.005	2	160.2
3	0.005	2	175.2
3	0.005	2	160.4
3	0.005	2	167.7
3	0.005	2	155.5
3	0.005	2	176.7
3	0.005	2	131.4
3	0.025	2	160.7
3	0.025	2	131.9
3	0.025	2	128.9
3	0.025	2	155.0
3	0.025	2	167.8
3	0.025	2	151.0
3	0.025	2	151.8
3	0.025	2	151.9
3	0.025	2	148.6
3	0.025	2	154.8
3	0.025	2	152.1
3	0.05	2	137.0
3	0.05	2	129.7
3	0.05	2	167.0
3	0.05	2	134.2
3	0.05	2	116.1
3	0.05	2	122.4
3	0.05	2	115.8
3	0.05	2	117.2
3	0.05	2	122.5
3	0.05	2	131.2
3	0.05	2	113.1
3	0.05	2	126.4
4	0	1	185.7
4	0	1	210.7
4	0	1	172.9
4	0	1	185.8
4	0	1	191.7
4	0	1	192.7
4	0	1	230.7
4	0	1	237.6
4	0	1	272.8

4	0	1	285.3
4	0	1	272.3
4	0	1	272.7
4	0	1	210.4
4	0	1	192.9
4	0.005	1	133.9
4	0.005	1	144.3
4	0.005	1	149.5
4	0.005	1	139.4
4	0.005	1	210.4
4	0.005	1	220.3
4	0.005	1	235.3
4	0.005	1	240.4
4	0.005	1	198.1
4	0.005	1	184.8
4	0.005	1	189.8
4	0.005	1	189.8
4	0.005	1	174.8
4	0.005	1	182.5
4	0.005	1	195.8
4	0.005	1	186.7
4	0.005	1	203.9
4	0.005	1	155.0
4	0.025	1	138.5
4	0.025	1	129.1
4	0.025	1	187.7
4	0.025	1	145.9
4	0.025	1	158.4
4	0.025	1	150.0
4	0.025	1	174.1
4	0.025	1	170.6
4	0.025	1	152.1
4	0.025	1	215.6
4	0.025	1	217.8
4	0.025	1	194.7
4	0.025	1	130.5
4	0.025	1	131.6
4	0.05	1	129.5
4	0.05	1	121.0
4	0.05	1	120.6
4	0.05	1	115.1
4	0.05	1	130.8
4	0.05	1	127.6
4	0.05	1	125.5
4	0.05	1	111.5
4	0.05	1	119.1
4	0.05	1	119.9
4	0.05	1	114.6
4	0.05	1	122.7
4	0.05	1	138.7

4	0.05	1	132.7
4	0	2	286.6
4	0	2	284.2
4	0	2	240.8
4	0	2	204.7
4	0	2	227.3
4	0	2	211.9
4	0	2	208.6
4	0	2	232.3
4	0	2	216.4
4	0	2	183.3
4	0	2	254.0
4	0.005	2	216.4
4	0.005	2	171.6
4	0.005	2	173.6
4	0.005	2	177.1
4	0.005	2	151.4
4	0.005	2	156.4
4	0.005	2	188.2
4	0.005	2	195.5
4	0.005	2	194.4
4	0.005	2	213.7
4	0.005	2	161.3
4	0.005	2	150.9
4	0.005	2	190.7
4	0.025	2	178.4
4	0.025	2	159.6
4	0.025	2	140.4
4	0.025	2	164.8
4	0.025	2	162.9
4	0.025	2	115.1
4	0.025	2	144.6
4	0.025	2	160.8
4	0.025	2	156.9
4	0.05	2	81.5
4	0.05	2	120.9
4	0.05	2	146.0
4	0.05	2	144.7
4	0.05	2	141.5
4	0.05	2	93.1
4	0.05	2	125.0
4	0.05	2	102.7
4	0.05	2	122.8
