# Supplementary material for: Sublethal cadmium exposure disrupts cardiac function and tissue structure in zebrafish larvae
Source: Front Physiol. 2026 Sep 21;17:1865285. doi: 10.3389/fphys.2026.1865285 (PMC13634864; doi:10.3389/fphys.2026.1865285)
Supplement: Supplementary file 2 [file Table2.pdf]

Table S2

Concentration	Heart rate at day 0, bpm, (mean ± standart deviation)	p-value (difference between Day 0 and Day 1)	Heart rate at day 1, bpm, (mean ± standart deviation)	p-value (difference between Day 1 and Day 2)	Heart rate at day 2, bpm, (mean ± standart deviation)
0.000 mg/l	159±13	0.0064	173±15	0.0218	184±20
0.005 mg/l	165±8	0.8404	166±22	0.5311	162±26
0.025 mg/l	139±19	0.4102	143±12	0.000	158±15
0.05 mg/l	111±11	0.0110	125±14	0.5187	128±18

p-value (difference between Day 2 and Day 3)	Heart rate at day 3, bpm, (mean ± standart deviation)	p-value (difference between Day 3 and Day 4)	Heart rate at day 4, bpm, (mean ± standart deviation)
0.0302	195±18	0.0001	226±36
0.7698	160±17	0.0006	183±28
0.0096	146±17	0.0337	160±26
0.1856	121±20	0.9004	122±15
