# Supplementary material for: Sublethal cadmium exposure disrupts cardiac function and tissue structure in zebrafish larvae
Source: Front Physiol. 2026 Sep 21;17:1865285. doi: 10.3389/fphys.2026.1865285 (PMC13634864; doi:10.3389/fphys.2026.1865285)
Supplement: Supplementary file 3 [file Table3.pdf]

Table S3

Heart wall thickness, µm		
Experimental group	Ventricle	Atrium
Control	7.31	2.19
	5.4	2.06
	8.94	2.96
	12.17	3.18
	11.44	3.16
	8.93	2.17
	7.5	2.13
	10.11	3.1
	6.12	3.23
	9.03	2.01
	8.72	3.65
	10.48	2.01
	6.84	2.32
	9.96	3.31
	8.31	2.06
	Ventricle	Atrium
0.05 mg/L	5.41	2.08
	7.34	2.05
	6.94	3.01
	7.38	1.95
	8.94	2.17
	8.35	3.31
	7.51	2.51
	5.13	2.05
	10.11	2.14
	9.01	2.86
	6.37	3.27
	5.39	2.05
	6.59	2.98
	7.57	2.75
	9.61	2.63
