# Supplementary material for: Sublethal cadmium exposure disrupts cardiac function and tissue structure in zebrafish larvae
Source: Front Physiol. 2026 Sep 21;17:1865285. doi: 10.3389/fphys.2026.1865285 (PMC13634864; doi:10.3389/fphys.2026.1865285)
Supplement: Supplementary file 4 [file Table4.pdf]

Table S4

Experimental group	Area of neuronal tissue on a section, μm^2	Area of neuronal degeneration regions, μm^2	Proportion of neuronal degeneration regions, %
0.000 mg/L	70311	0	0.00
	9674	0	0.00
	68511	0	0.00
	53694	0	0.00
	46799	0	0.00
	22365	0	0.00
	67869	0	0.00
	76151	0	0.00
	63452	0	0.00
	68568	0	0.00
	79508	0	0.00
	76267	0	0.00
	49163	0	0.00
	40362	324	0.80
	61299	0	0.00
0.050 mg/L	67600	363	0.54
	53231	686	1.29
	40422	2995	7.41
	63356	9001	14.21
	49050	3946	8.04
	65589	5225	7.97
	63338	8949	14.13
	62886	5741	9.13
	58682	1337	2.28
	46823	0	0.00
	52013	324	0.62
	93425	6352	6.80
	54329	3182	5.86
	49915	0	0.00
	78964	1321	1.67
0.025 mg/L	44646	0	0.00
	88402	1414	1.60
	92488	0	0.00
	104154	0	0.00
	47875	0	0.00
	82169	0	0.00
	64599	0	0.00
	45967	0	0.00
	77802	779	1.00
	73684	0	0.00
	35062	0	0.00
	42824	0	0.00
