# Supplementary material for: Sublethal cadmium exposure disrupts cardiac function and tissue structure in zebrafish larvae
Source: Front Physiol. 2026 Sep 21;17:1865285. doi: 10.3389/fphys.2026.1865285 (PMC13634864; doi:10.3389/fphys.2026.1865285)
Supplement: Supplementary file 5 [file Table5.pdf]

Table S5. Descriptive statistics and one-way ANOVA by session

Day	Repetion	Concentration, mg/L	n	Mean heart rate, bpm	SD, bpm	ANOVA F	df (between groups)	df (within groups)	ANOVA p
0	1	0	14	159.2	13.4	29.125	3	47	<0.0001
		0.005	11	165.2	8.3				
		0.025	17	139.2	19.2				
		0.05	9	110.6	11.4				
1	1	0	20	170.4	14.8	22.993	3	71	<0.0001
		0.005	24	165	13.8				
		0.025	24	143.8	11.9				
		0.05	7	133.6	16.8				
	2	0	6	183.1	14.6	17.454	3	36	<0.0001
		0.005	12	170	34.2				
		0.025	10	140.4	10.8				
		0.05	12	120	8.7				
2	1	0	14	174.5	9.6	16.306	3	54	<0.0001
		0.005	13	167.8	29.7				
		0.025	17	160.1	12.5				
		0.05	14	127.1	21.7				
	2	0	14	195.2	22.2	29.314	3	49	<0.0001
		0.005	12	157.2	20.2				
		0.025	14	154.4	17.4				
		0.05	13	129.4	12.8				
3	1	0	15	189.8	18.9	39.09	3	53	<0.0001
		0.005	13	168.3	17.5				
		0.025	13	142.1	20.2				
		0.05	16	116.7	21.9				
	2	0	17	201.1	16.6	64.805	3	51	<0.0001
		0.005	15	154.7	14.8				
		0.025	11	150.4	11.3				
		0.05	12	127.7	14.6				
4	1	0	14	222.4	39.2	27.238	3	56	<0.0001
		0.005	18	185.3	31.4				
		0.025	14	164	30.3				
		0.05	14	123.5	7.7				
	2	0	11	231.8	32.6	36.555	3	38	<0.0001
		0.005	13	180.1	21.9				
		0.025	9	153.7	18.2				
		0.05	9	119.8	23.1				

Table S5 (continued). Descriptive statistics

Day	Repetition	Experimental group 1 (EG 1)	Experimental group 2 (EG 2)	n (EG 1)	Mean (EG 1), bpm	Standart deviation (EG 1)	n (EG 2)	Mean (EG 2), bpm	Standart deviation (EG 1)	Mean difference (EG 2 − EG 1)	Tukey HSD p-adj
0	1	0.000 mg/L	0.005 mg/L	14	159.2	13.4	11	165.2	8.3	6.01	0.736
0	1	0.000 mg/L	0.025 mg/L	14	159.2	13.4	17	139.2	19.2	-19.97	0.0023
0	1	0.000 mg/L	0.050 mg/L	14	159.2	13.4	9	110.6	11.4	-48.58	<0.0001
0	1	0.005 mg/L	0.025 mg/L	11	165.2	8.3	17	139.2	19.2	-25.98	0.0002
0	1	0.005 mg/L	0.050 mg/L	11	165.2	8.3	9	110.6	11.4	-54.59	<0.0001
0	1	0.025 mg/L	0.050 mg/L	17	139.2	19.2	9	110.6	11.4	-28.61	0.0001
1	1	0.000 mg/L	0.005 mg/L	20	170.4	14.8	24	165	13.8	-5.48	0.5581
1	1	0.000 mg/L	0.025 mg/L	20	170.4	14.8	24	143.8	11.9	-26.61	<0.0001
1	1	0.000 mg/L	0.050 mg/L	20	170.4	14.8	7	133.6	16.8	-36.78	<0.0001
1	1	0.005 mg/L	0.025 mg/L	24	165	13.8	24	143.8	11.9	-21.13	<0.0001
1	1	0.005 mg/L	0.050 mg/L	24	165	13.8	7	133.6	16.8	-31.3	<0.0001
1	1	0.025 mg/L	0.050 mg/L	24	143.8	11.9	7	133.6	16.8	-10.17	0.3226
1	2	0.000 mg/L	0.005 mg/L	6	183.1	14.6	12	170	34.2	-13.16	0.5973
1	2	0.000 mg/L	0.025 mg/L	6	183.1	14.6	10	140.4	10.8	-42.7	0.0019
1	2	0.000 mg/L	0.050 mg/L	6	183.1	14.6	12	120	8.7	-63.1	<0.0001
1	2	0.005 mg/L	0.025 mg/L	12	170	34.2	10	140.4	10.8	-29.54	0.0116
1	2	0.005 mg/L	0.050 mg/L	12	170	34.2	12	120	8.7	-49.94	<0.0001
1	2	0.025 mg/L	0.050 mg/L	10	140.4	10.8	12	120	8.7	-20.4	0.1242
2	1	0.000 mg/L	0.005 mg/L	14	174.5	9.6	13	167.8	29.7	-6.65	0.8115
2	1	0.000 mg/L	0.025 mg/L	14	174.5	9.6	17	160.1	12.5	-14.41	0.1816
2	1	0.000 mg/L	0.050 mg/L	14	174.5	9.6	14	127.1	21.7	-47.41	<0.0001
2	1	0.005 mg/L	0.025 mg/L	13	167.8	29.7	17	160.1	12.5	-7.77	0.7007
2	1	0.005 mg/L	0.050 mg/L	13	167.8	29.7	14	127.1	21.7	-40.76	<0.0001
2	1	0.025 mg/L	0.050 mg/L	17	160.1	12.5	14	127.1	21.7	-33	0.0001
2	2	0.000 mg/L	0.005 mg/L	14	195.2	22.2	12	157.2	20.2	-38.04	<0.0001

2	2	0.000 mg/L	0.025 mg/L	14	195.2	22.2	14	154.4	17.4	-40.82	<0.0001
2	2	0.000 mg/L	0.050 mg/L	14	195.2	22.2	13	129.4	12.8	-65.85	<0.0001
2	2	0.005 mg/L	0.025 mg/L	12	157.2	20.2	14	154.4	17.4	-2.78	0.9808
2	2	0.005 mg/L	0.050 mg/L	12	157.2	20.2	13	129.4	12.8	-27.81	0.0025
2	2	0.025 mg/L	0.050 mg/L	14	154.4	17.4	13	129.4	12.8	-25.03	0.0052
3	1	0.000 mg/L	0.005 mg/L	15	189.8	18.9	13	168.3	17.5	-21.56	0.0288
3	1	0.000 mg/L	0.025 mg/L	15	189.8	18.9	13	142.1	20.2	-47.68	<0.0001
3	1	0.000 mg/L	0.050 mg/L	15	189.8	18.9	16	116.7	21.9	-73.08	<0.0001
3	1	0.005 mg/L	0.025 mg/L	13	168.3	17.5	13	142.1	20.2	-26.12	0.0076
3	1	0.005 mg/L	0.050 mg/L	13	168.3	17.5	16	116.7	21.9	-51.52	<0.0001
3	1	0.025 mg/L	0.050 mg/L	13	142.1	20.2	16	116.7	21.9	-25.4	0.0061
3	2	0.000 mg/L	0.005 mg/L	17	201.1	16.6	15	154.7	14.8	-46.42	<0.0001
3	2	0.000 mg/L	0.025 mg/L	17	201.1	16.6	11	150.4	11.3	-50.67	<0.0001
3	2	0.000 mg/L	0.050 mg/L	17	201.1	16.6	12	127.7	14.6	-73.38	<0.0001
3	2	0.005 mg/L	0.025 mg/L	15	154.7	14.8	11	150.4	11.3	-4.25	0.8867
3	2	0.005 mg/L	0.050 mg/L	15	154.7	14.8	12	127.7	14.6	-26.95	0.0001
3	2	0.025 mg/L	0.050 mg/L	11	150.4	11.3	12	127.7	14.6	-22.7	0.003
4	1	0.000 mg/L	0.005 mg/L	14	222.4	39.2	18	185.3	31.4	-37.17	0.0048
4	1	0.000 mg/L	0.025 mg/L	14	222.4	39.2	14	164	30.3	-58.41	<0.0001
4	1	0.000 mg/L	0.050 mg/L	14	222.4	39.2	14	123.5	7.7	-98.91	<0.0001
4	1	0.005 mg/L	0.025 mg/L	18	185.3	31.4	14	164	30.3	-21.24	0.1982
4	1	0.005 mg/L	0.050 mg/L	18	185.3	31.4	14	123.5	7.7	-61.74	<0.0001
4	1	0.025 mg/L	0.050 mg/L	14	164	30.3	14	123.5	7.7	-40.51	0.0036
4	2	0.000 mg/L	0.005 mg/L	11	231.8	32.6	13	180.1	21.9	-51.73	0.0001
4	2	0.000 mg/L	0.025 mg/L	11	231.8	32.6	9	153.7	18.2	-78.09	<0.0001
4	2	0.000 mg/L	0.050 mg/L	11	231.8	32.6	9	119.8	23.1	-112.02	<0.0001
4	2	0.005 mg/L	0.025 mg/L	13	180.1	21.9	9	153.7	18.2	-26.36	0.0842
4	2	0.005 mg/L	0.050 mg/L	13	180.1	21.9	9	119.8	23.1	-60.29	<0.0001
4	2	0.025 mg/L	0.050 mg/L	9	153.7	18.2	9	119.8	23.1	-33.93	0.0298
