# Supplementary material for: Sublethal cadmium exposure disrupts cardiac function and tissue structure in zebrafish larvae
Source: Front Physiol. 2026 Sep 21;17:1865285. doi: 10.3389/fphys.2026.1865285 (PMC13634864; doi:10.3389/fphys.2026.1865285)
Supplement: Supplementary file 6 [file Table6.pdf]

Table S6. Spearman rank correlation between nominal cadmium concentration and heart rate

Day	Repetition	Total n	n (0.000 mg/L)	n (0.005 mg/L)	n (0.025 mg/L)	n (0.050 mg/L)	Spearman rho	p-value
0	1	51	14	11	17	9	-0.683	<0.0001
1	1	75	20	24	24	7	-0.674	<0.0001
1	2	40	6	12	10	12	-0.883	<0.0001
2	1	58	14	13	17	14	-0.687	<0.0001
2	2	53	14	12	14	13	-0.79	<0.0001
3	1	57	15	13	13	16	-0.858	<0.0001
3	2	55	17	15	11	12	-0.859	<0.0001
4	1	60	14	18	14	14	-0.78	<0.0001
4	2	42	11	13	9	9	-0.881	<0.0001
