# Supplementary material for: Sublethal cadmium exposure disrupts cardiac function and tissue structure in zebrafish larvae
Source: Front Physiol. 2026 Sep 21;17:1865285. doi: 10.3389/fphys.2026.1865285 (PMC13634864; doi:10.3389/fphys.2026.1865285)
Supplement: Supplementary file 7 [file Table7.pdf]

Table S7. Developmental morphometry

Day	Concentration, mg/L	Body length, mm (mean ± standart deviation)	Pericardial area, mm² (mean ± standart deviation)
0	0.000	3.62 ± 0.15	0.013 ± 0.002
0	0.005	3.50 ± 0.12	0.015 ± 0.004
0	0.025	3.62 ± 0.12	0.014 ± 0.003
0	0.050	3.66 ± 0.10	0.015 ± 0.004
ANOVA p-value		0.118	0.8232
4	0.000	4.72 ± 0.15	0.047 ± 0.011
4	0.005	4.76 ± 0.09	0.056 ± 0.018
4	0.025	4.55 ± 0.09	0.060 ± 0.005
4	0.050	4.53 ± 0.18	0.051 ± 0.019
ANOVA p-value		0.2354	0.8337
